# The AKT-independent MET–V-ATPase–MTOR axis suppresses liver cancer vaccination

**DOI:** 10.1038/s41392-020-0179-x

**Published:** 2020-08-07

**Authors:** Xing Huang, Xingyuan Xu, Xun Wang, Tianyu Tang, Enliang Li, Xiaozhen Zhang, Jian Xu, Hang Shen, Chengxiang Guo, Tao Xu, Jianhong Ren, Xueli Bai, Tingbo Liang

**Affiliations:** 1 Zhejiang Provincial Key Laboratory of Pancreatic Disease, The First Affiliated Hospital, School of Medicine, Zhejiang University, Hangzhou, 310003 Zhejiang China; 2 Department of Hepatobiliary and Pancreatic Surgery, The First Affiliated Hospital, School of Medicine, Zhejiang University, Hangzhou, 310003 Zhejiang China; 3 Innovation Center for the Study of Pancreatic Diseases of Zhejiang Province, Hangzhou, 310003 Zhejiang China; 4 The Key Laboratory of Developmental Genes and Human Disease, Institute of Life Sciences, Southeast University, Nanjing, 210096 Jiangsu China; 5 Suzhou BioNovoGene Metabolomics Platform, Suzhou, 215000 Jiangsu China

**Keywords:** Gastrointestinal cancer, Tumour immunology, Vaccines

## Abstract

Despite recent progress in hepatitis treatment, there have been no significant advances in the development of liver cancer vaccines in recent years. In this study, we investigated the regulatory effect and potential mechanism of hepatocyte growth factor receptor (MET, also known as HGFR) on tumor vaccinations for liver cancer in mice. Herein, we demonstrate that MET expression is significantly associated with the immunogenicity of liver cancer in mice and humans, and that MET depletion dramatically enhances the protective efficacy of chemotherapy-based anti-liver cancer vaccination. Mechanistically, MET repressed liver cancer immunogenicity independent of the traditional PI3K–AKT cascade, and MET interacted with vacuolar ATP synthase (V-ATPase) and mediated the activation of mammalian target of rapamycin (MTOR), thus suppressing liver cancer immunogenicity. The efficacy of chemotherapy-based liver cancer vaccination was markedly enhanced by targeting the MET–V-ATPase–MTOR axis, highlighting a translational strategy for identifying MET-associated drug candidates for cancer prevention.

## Introduction

The early diagnosis and therapeutic management of liver cancer remain challenging tasks due to the high fatality rate of this cancer.^1–3^ Tumor vaccination is considered an ideal approach for liver cancer prevention and adjuvant therapy;^4,5^ however, no liver cancer vaccines have been developed to date. Further research on liver cancer vaccines has been primarily hindered by limited knowledge of the molecular mechanisms that drive the suppression of liver cancer immunogenicity. Herein, we focus on MET, an important receptor tyrosine kinase (RTK) and the receptor of hepatocyte growth factor (HGF).^6–8^ After binding to HGF, MET is activated via dimerization and autophosphorylation to initiate downstream signaling pathways.^8,9^ Ample evidence has shown the significance of MET in liver development and oncogenic transformation, as well as its role in therapeutic resistance;^10–13^ thus, the current study was aimed at determining the effect of MET on liver cancer immunogenicity and the protective efficacy of liver cancer vaccination.

The mechanistic (mammalian) target of rapamycin complex (MTORC) is critical for sensing multiple variables related to metabolism,^14^ and thus, it has been recognized as a pivotal factor for controlling metabolite-dependent cell growth.^15^ Indeed, hyperactive MTOR signaling is frequently observed in cancer pathology.^16,17^ Many factors regulate MTOR activation in different physiological and pathological conditions, including but not limited to KLHL22, SAMTOR, and Sestrin2;^16,18–21^ however, the potential influences of MTOR on tumor vaccination have rarely been characterized. Herein, we identify an AKT-independent MET–MTOR signaling pathway useful for determining the efficacy of liver cancer vaccination. We found that MET serves as a central component of the MTOR complex, independent of the PI3K–AKT cascade, and participates in MTOR activation by interacting with V-ATPase. We also demonstrate that the newly discovered MET–V-ATPase–MTOR signaling pathway more strongly suppresses liver cancer immunogenicity than does the traditional MET–AKT–MTOR pathway.

## Results

### MET suppresses anti-liver cancer immunity in mice and humans

**Fig. 1:**
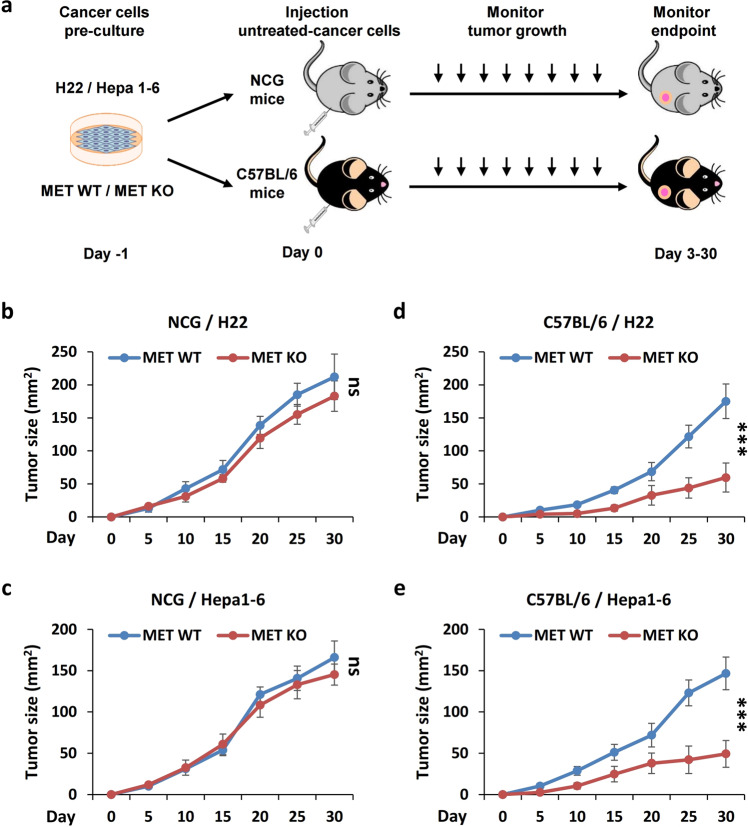
MET contributes to liver cancer growth in an immune system-dependent manner. **a** Strategy for investigating the immunity-related effects of MET on liver cancer growth. **b**, **c** Impact of MET depletion on liver cancer growth in immunodeficient mice. NCG mice were individually transplanted *s.c*. with wild-type (WT) and MET-deficient (MET-KO) H22 cells (**b**) or Hepa1-6 cells (**c**), respectively. Tumor size was monitored at the indicated times. **d**, **e** Impact of MET depletion on liver cancer growth in the immunocompetent mice. C57BL/6 mice were individually transplanted *s.c*. with WT and MET-KO H22 cells (**d**) or Hepa1-6 cells (**e**), respectively. Tumor size was monitored at the indicated times. *p-Value < 0.05, **p-value < 0.01, ***p-value < 0.001, ns (nonsignificant), compared to the indicated groups; *n* = 10 per group.

To determine whether MET regulates liver cancer growth by disrupting immune surveillance, we constructed two xenograft models by inoculating wild-type (WT) and MET-depleted (*Met*-KO) H22 (Supplementary Fig. S1a) and Hepa1-6 (Supplementary Fig. S1b) murine liver cancer cell lines in severely immunodeficient NOD-*Prkdc*^*em26Cd52*^*Il2rg*^*em26Cd22*^ (NCG) mice and in immunocompetent C57BL/6 mice, respectively (Fig. 1a). No significant difference was observed in the cancer growth of the *Met*-KO H22 cells and the WT control cells in the NCG mice (Fig. 1b). Moreover, similar phenotypes were also observed in the same experimental settings using another liver cancer cell line, Hepa1-6 cells (Fig. 1c). In contrast, MET deficiency markedly inhibited the tumor growth in the C57BL/6 mice inoculated with either H22 cells (Fig. 1d) or Hepa1-6 cells (Fig. 1e).

**Fig. 2:**
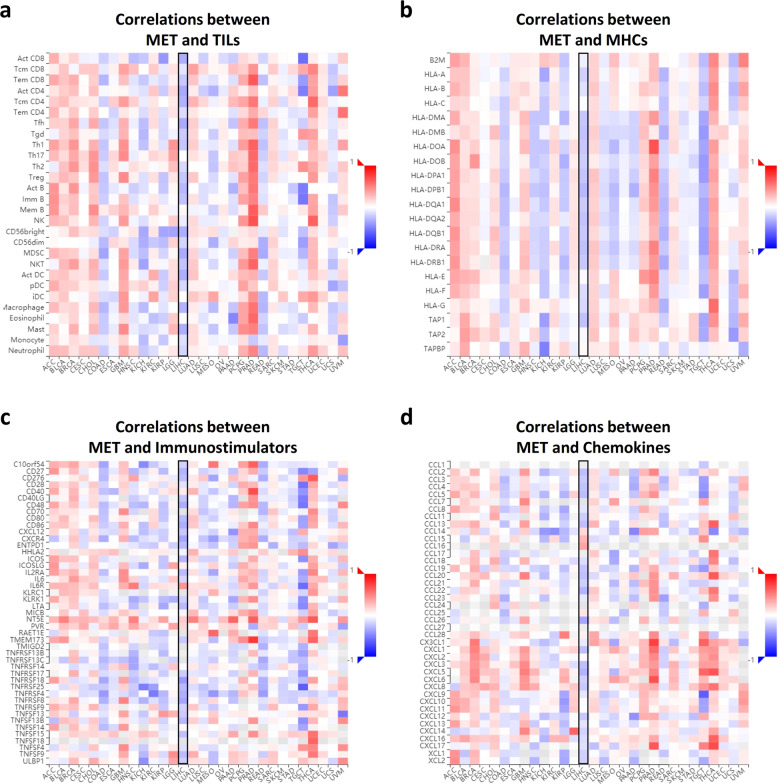
MET correlates negatively with immune signatures in liver cancer clinical samples. **a** Spearman correlations between the abundance of MET expression and the immune-related signatures of multiple tumor-infiltrating lymphocytes (TILs) across human cancers. For each cancer type, the relative abundance of TILs was inferred by using gene set variation analysis based on the gene expression profile (TCGA). **b**–**d** Spearman correlations of the abundance of MET expression and multiple immune regulatory molecule levels. The relationship between MET and multiple MHCs (**b**), immunostimulators (**c**), and chemokines (**d**) across human cancers (TCGA) are shown as indicated. The detailed readouts of the LIHC cells are highlighted in the black boxes.

To better understand the clinical association between MET and cancer immunity, we utilized the Tumor and Immune System Interaction Database (TISIDB), a public web portal based on The Cancer Genome Atlas (TCGA), to analyze the relevance of MET to multiple immune features across 30 cancer types. Consistent with the mouse data, the expression levels of MET in clinical samples exhibited a negative correlation with the relative abundance of most tumor-infiltrating lymphocytes (TILs) (Fig. 2a), major histocompatibility complexes (MHCs) (Fig. 2b), immunomodulators (Fig. 2c), and chemokines (Fig. 2d) in liver hepatocellular carcinoma (LIHC). These multidimensional profiling results strongly suggested an active role of MET in controlling liver cancer immunogenicity.

### MET depletion facilitates liver cancer vaccination independent of AKT activity

**Fig. 3:**
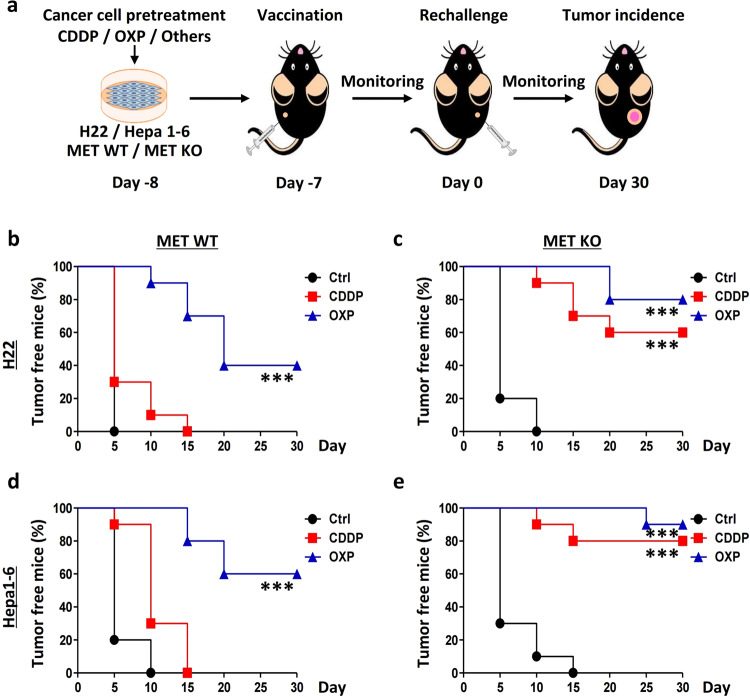
MET suppresses the protective efficacy of chemotherapy-based liver cancer vaccination. **a** Strategy for evaluating the potential effects of MET in chemotherapy-based liver cancer vaccination. **b**–**e** MET deficiency enhanced the protective efficacy of chemotherapy-based liver cancer vaccination. Wild-type (WT) and MET-deficient (MET-KO) H22 cells (**b**, **c**) and Hepa1-6 cells (**d**, **e**) were individually treated with cisplatin (CDDP), oxaliplatin (OXP), or a vehicle control (Ctrl), respectively, and then subcutaneously (*s.c*.) injected into the left flank of C57BL/6 mice. One week later, all the mice were rechallenged with homologous untreated WT cells in the right flank. The tumor incidence is reported as Kaplan–Meier curves. Significance was determined by the means calculated with a likelihood ratio test. *p-Value < 0.05, **p-value < 0.01, ***p-value < 0.001, and ns (nonsignificant), compared to the indicated groups; *n* = 10 per group.

Oxaliplatin (OXP), but not cisplatin (CDDP), has been reported as a viable treatment that serves as a tumor vaccination in several cancer types, as observed by OXP induction of the immunogenic death of cancer cells and activation of tumor immunogenicity.^22,23^ In the present study, using a pre-setup protocol (Fig. 3a), we observed that vaccination could protect 40% of the WT H22 cells (tumor-free) that were injected into mice which were pre-challenged by OXP-treated H22 cells (Fig. 3b). Intriguingly, 80% of the mice were protected by the vaccination of OXP-treated MET-depleted H22 cells. In addition, CDDP practically induced cancer vaccination in the absence of MET (from 0 to 60% of the mice) (Fig. 3c). Similarly, the WT Hepa1-6 cells treated with OXP conferred 60% protection to the mice, and this rate was increased to 90% for the mice pre-challenged by the *Met*-KO cells; furthermore, 80% protection was observed in the mice using the CDDP-treated *Met*-KO cells (Fig. 3d, e).

**Fig. 4:**
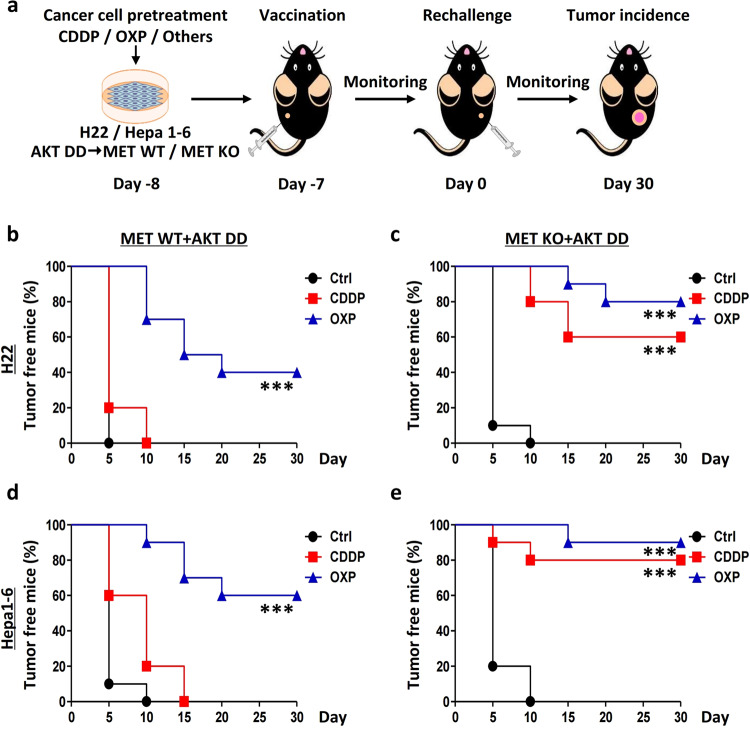
MET depletion enhances liver cancer vaccination in an AKT-independent manner. **a** Strategy for investigating the dependency of AKT in MET-controlled liver cancer vaccination. **b**–**e** AKT activation was not able to repress MET deficiency-enhanced liver cancer immunogenicity. Wild-type (WT) and MET-deficient (MET-KO) H22 cells (**b**, **c**) and Hepa1-6 cells (**d**, **e**) were individually transfected with the Akt T308D/S473D (AKT-DD) mutant, subsequently treated with cisplatin (CDDP), oxaliplatin (OXP), or vehicle control (Ctrl), respectively, and then subcutaneously (*s.c*.) injected into the left flank of C57BL/6 mice. One week later, all the mice were rechallenged with homologous untreated WT cells in the right flank. The tumor incidence is reported as Kaplan–Meier curves. Significance was determined by the means calculated with a likelihood ratio test. *p-Value < 0.05, **p-value < 0.01, ***p-value < 0.001, ns (nonsignificant), compared to the indicated groups; *n* = 10 per group.

MET, a classical receptor tyrosine kinase (RTK), exerts its functions mainly through its pivotal downstream executor in the phosphatidylinositol-4,5-bisphosphate 3-kinase (PI3K)–AKT cascade.^24^ To determine whether MET-controlled liver cancer immunogenicity is mediated by the AKT signaling pathway, we introduced the AKT constitutively activated mutant T308D/S473D (AKT-DD) and repeated the experiments described above (Fig. 4a). Unexpectedly, AKT activation did not repress MET deficiency-enhanced liver cancer immunogenicity. Briefly, the AKT-DD mutant failed to reduce the protective efficacy of the OXP- or CDDP-based vaccination using the WT or MET-depleted H22 cells (Fig. 4b, c) or the Hepa1-6 cells (Fig. 4d, e). These results strongly indicate that MET regulates liver cancer immunogenicity in an AKT-independent manner.

### V-ATPase is a novel target of MET in liver cancer

**Fig. 5:**
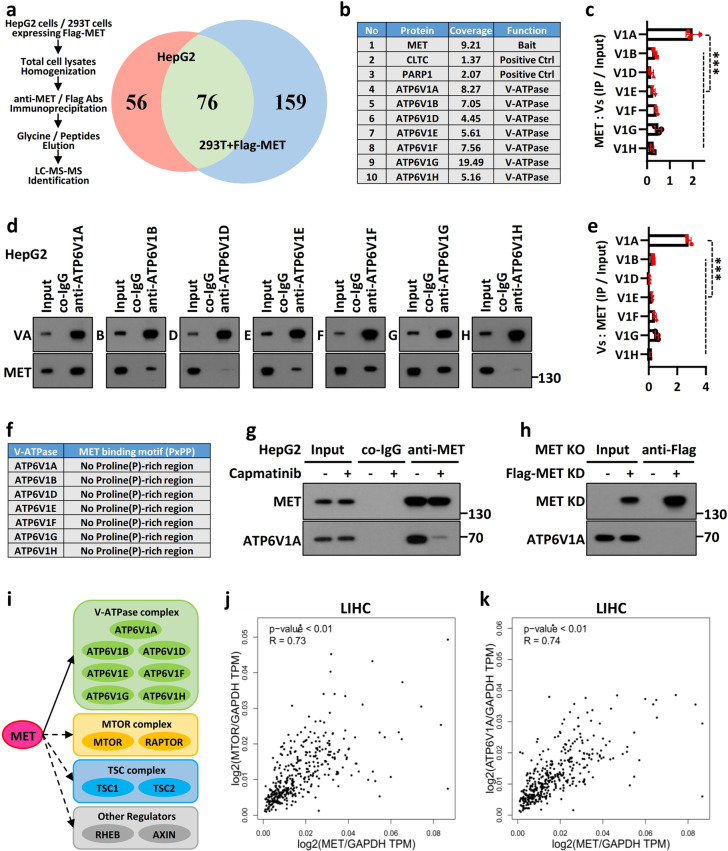
V-ATPase was identified as a novel protein interacting with MET in the MTOR complex. **a** Mass spectrometric analysis of MET immunoprecipitates. HepG2 cells and 293T cells expressing Flag-MET were individually subjected to immunoprecipitation, and potential MET-interacting proteins interactors were identified by LC–MS/MS. Left: workflow; right: Venn diagram. **b** Representative MET-interaction candidates, including positive controls, are listed as indicated. **c** MET interacts with the lysosomal V-ATPase complex. Lysates from HepG2 cells were subjected to immunoprecipitation with anti-MET antibody or co-IgG. The ratio of each subunit in the V-ATPase complex that was immunoprecipitated with MET is shown as indicated. **d** MET binds to the ATP6V1A subunit in the V-ATPase complex. Lysates from HepG2 cells were subjected to immunoprecipitation with antibodies against the subunits of V-ATPase or co-IgG. **e** The ratio of MET to each subunit of the V-ATPase complex that was immunoprecipitated is shown as indicated. **f** MET interacts with the V-ATPase complex in a noncanonical manner. Detailed sequence analyses of the V-ATPase complex showing no conserved MET-binding PxPP motif. **g** Inhibition of MET phosphorylation disrupts the MET–V-ATPase interaction. HepG2 cells were individually treated with or without 50 nM capmatinib for 2 h. Cell lysates were subjected to immunoprecipitation and immunoblotting with the indicated antibodies. **h** MET-KD mutant lost the ability to interact with V-ATPase. MET-KO HepG2 cells were individually transfected with the MET-KD mutant or vector control for 36 h and then subjected to an immunoprecipitation assay. **i** MET is a critical component of the MTOR complex. The schematic landscape of MET-interacting proteins was adapted from the MS spectra, the FpClass and STRING databases. Solid arrows denote the proteins that were identified with MS, and the dashed lines indicate the potential interactions predicted through database analysis. **j** MET correlates with MTOR in LIHC (TCGA, *n* = 369). **k** MET correlates with ATP6V1A in LIHC (TCGA, *n* = 369). GAPDH was applied as a control in the correlation analysis to normalize gene expression at the RNA level.

To identify the potential targets of MET in the repression of cancer immunogenicity, we first sought to identify MET-interacting proteins using an affinity purification approach. The extracts from HepG2 cells and 293T cells expressing a Flag-tagged MET protein (Flag-MET) were individually immunoprecipitated and subsequently analyzed using tandem mass spectrometry (LC–MS/MS). The candidate proteins (those specifically bound to the endogenous MET and exogenous Flag-MET-immobilized beads, but not to the isotype control) were selected, and the data were overlapped in a Venn diagram (Fig. 5a). Intriguingly, almost the entire V1 domain of the lysosomal vacuolar H^+^-adenosine triphosphatase ATPase (V-ATPase) complex, including subunits A, B, D, E, F, G, and H (but not C), was immunoprecipitated with MET (Fig. 5b). Concomitantly, MET itself and proteins previously reported to interact with it, such as clathrin heavy chain (CLTC) and poly (ADP-ribose) polymerase 1 (PARP1),^25,26^ were also identified and served as positive controls.

The V-ATPase complex, an integral component of the MTOR machinery, is essential for amino acid activation of MTORC1 through an inside-out mechanism.^18^ To identify the subunit in the V-ATPase complex that dominates the interaction with MET, we performed bidirectional immunoprecipitation experiments with antibodies against MET (Fig. 5c) or individual subunits of the V-ATPase complex (Fig. 5d, e) and observed that ATP6V1A was the most highly enriched subunit among the MET interaction partners. A conserved proline-rich region (PxPP) is essential for substrate binding to MET^27^ and the indirect interaction mediated by typical adaptor proteins, such as growth factor receptor-bound protein 2 (GRB2) and GRB2-associated-binding protein 1 (GAB1).^27–29^ To determine whether the V-ATPase complex could interact with MET via the PxPP motif, we retrieved the sequences of the V-ATPase complex. Notably, none of the subunits of the V-ATPase complex contained a PxPP or analogous motif (Fig. 5f), including ATP6V1A (Supplementary Fig. S2a). Moreover, the immunoprecipitation assays demonstrated no association between the V-ATPase complex and GRB2 or GAB1 (Supplementary Fig. S2b, c). These findings indicated that MET might interact with the lysosomal V-ATPase complex noncanonically.

No evidence suggests that proteins interact with the V-ATPase in a phosphorylation-dependent manner. Because MET is a tyrosine kinase activated by HGF, its actions are generally induced upon its phosphorylation. Capmatinib is a highly selective small-molecule inhibitor of MET with picomolar potency and more than 10,000-fold selectivity for MET over a large panel of other human kinases.^30^ Therefore, to ascertain the association of MET phosphorylation with V-ATPase, we examined the effects of capmatinib on the MET–V-ATPase interaction and found that capmatinib nearly completely inhibited the MET–ATP6V1A association (Fig. 5g). Furthermore, we used the phosphorylation-associated kinase-dead mutant of MET (MET-KD, all five pivotal tyrosine residues mutated to phenylalanine) for binding analysis and observed that the MET-KD mutant lost its ability to interact with ATP6V1A (Fig. 5h). These results suggest that MET binds to ATP6V1A in a phosphorylation-dependent manner.

MET is predominantly classified as a plasma membrane-bound receptor, whereas V-ATPase is mostly found in endosomes and lysosomes, but HGF stimulation-induced phosphorylation led to significantly MET intracellular distribution via clathrin-mediated endocytosis.^31^ Therefore, we also investigated the location of the MET–V-ATPase interaction through subcellular fractionation. We utilized the selective clathrin inhibitor Pitstop 2 to inhibit clathrin-mediated endocytosis and observed the inhibition of both HGF-stimulated MET lysosomal translocation (Fig. S3a) and the MET–ATP6V1A interaction (Supplementary Fig. S3b). Furthermore, lysosomal fraction-based immunoprecipitation assays also established that MET binds to V-ATPase on lysosomes through endocytosis (Supplementary Fig. S3c).

Additionally, the bioinformatics prediction of potential MET-interacting proteins from the FpClass and STRING 10.5 databases suggested that MET was associated with the mammalian target of rapamycin (MTOR)-centric lysosomal sensing machinery (Fig. 5i). Further analyses revealed that MET was positively correlated with the MTOR levels (*R* = 0.73, *p* < 0.01) (Fig. 5j) and ATP6V1A levels (*R* = 0.74, *p* < 0.01) (Fig. 5k) in clinical cases of LIHC.

### MET suppresses liver cancer vaccination through V-ATPase–MTOR signaling

**Fig. 6:**
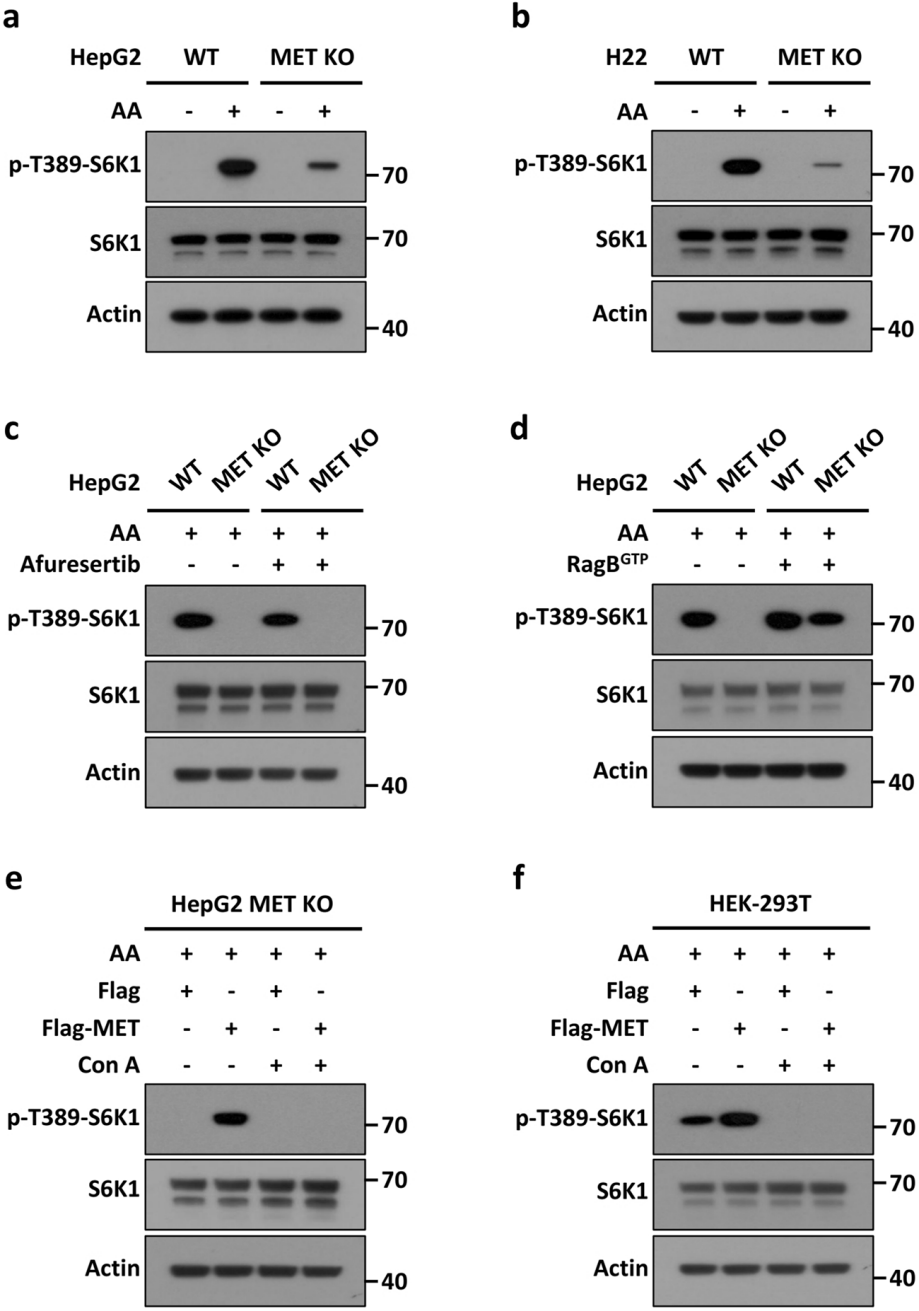
MET mediated MTOR activation through V-ATPase, not AKT. **a**, **b** MET is required for amino acid-stimulated MTOR activation. WT and MET-KO HepG2 (**a**) and H22 (**b**) cells were individually deprived of amino acids for 90 min and then stimulated with or without amino acids for 45 min. Cell lysates were subjected to immunoblotting with the indicated antibodies. **c** MET participates in amino acid-stimulated MTOR activation in an AKT-independent manner. WT and MET-KO HepG2 cells were individually treated with or without 1 μM afuresertib for 12 h, deprived of amino acids for 90 min, and subsequently stimulated with amino acids for 45 min. Cell lysates were subjected to immunoblotting with the indicated antibodies. **d** MET functions upstream of the Rag GTPase complex upon MTOR signaling. WT and MET-KO HepG2 cells were individually transfected with or without the RagB Q99L (RagB^GTP^) mutant for 36 h, deprived of amino acids for 90 min, and subsequently stimulated with amino acids for 45 min. Cell lysates were subjected to immunoblotting with the indicated antibodies. **e**, **f** MET controlled amino acid-stimulated MTOR activation via V-ATPase. MET-KO HepG2 (**e**) and HEK293T (**f**) cells were individually transfected with Flag-MET or a vector control for 36 h, starved for 90 min in the presence of 5 μM concanamycin A (Con A) or a vehicle, and subsequently stimulated with amino acids for 45 min. Cell lysates were subjected to immunoblotting with the indicated antibodies.

MTOR phosphorylates a series of growth- and metabolism-associated substrates, among which the best-characterized target is ribosomal protein S6 (RPS6) kinase 1 (S6K1).^32^ MTOR phosphorylates S6K1 at Thr389, and activated S6K1 further phosphorylates RPS6 to regulate protein translation, synthesis, and metabolism.^15^ To investigate the role of MET in MTOR activation, we used WT and *Met*-KO HepG2 cells and observed that the amino acid-enhanced phosphorylation of S6K1 at Thr389 was repressed in MET-KO cells (Fig. 6a). A similar phenomenon was also observed in WT and MET-KO H22 cells (Fig. 6b). We further validated this phenomenon in tissue and non-liver cancer cells and found that the amino acid-induced phosphorylation of S6K1 was also significantly repressed in mice expressing a liver-specific Met KO (liver conditional Met-knockout mouse) (Supplementary Fig. S4a) and MET-depleted B16 cells (mouse melanoma) (Supplementary Fig. S4b). These results indicated that MET, in general, regulates amino acid-induced MTOR activation.

To exclude the possibility that AKT is involved in these effects, we used the AKT-specific small molecule inhibitor afuresertib, and the results indicated that MET participated in amino acid-stimulated MTOR activation independent of AKT, as indicated by MET regulating MTOR activation independent of AKT activation (Fig. 6c). Following an amino acid-sensitive and ATP hydrolysis-dependent manner, the multicomponent V-ATPase complex established the lysosomal proton gradient and interacted with Ragulator (a scaffolding complex that anchors the Rag GTPases to the lysosomal surface), which in turn promoted the translocation and activation of MTOR.^18,33^ Forced expression of constitutively active the Rag GTPase mutant Q99L (RagB^GTP^) rescued S6K1 phosphorylation at Thr389, that had been abolished due to MET depletion (Fig. 6d). Finally, we observed that the V-ATPase-specific inhibitor concanamycin A (Con A) completely abrogated the MET-induced S6K1 phosphorylation in the MET-rescued HepG2 cells (Fig. 6e) and MET-expressing 293T cells with constitutively activated AKT (Fig. 6f). Collectively, these results indicate that MET is essential for regulating MTOR activity via V-ATPase and for activating upstream the Rag GTPase complex; however, this MET action is independent of AKT signaling.

**Fig. 7:**
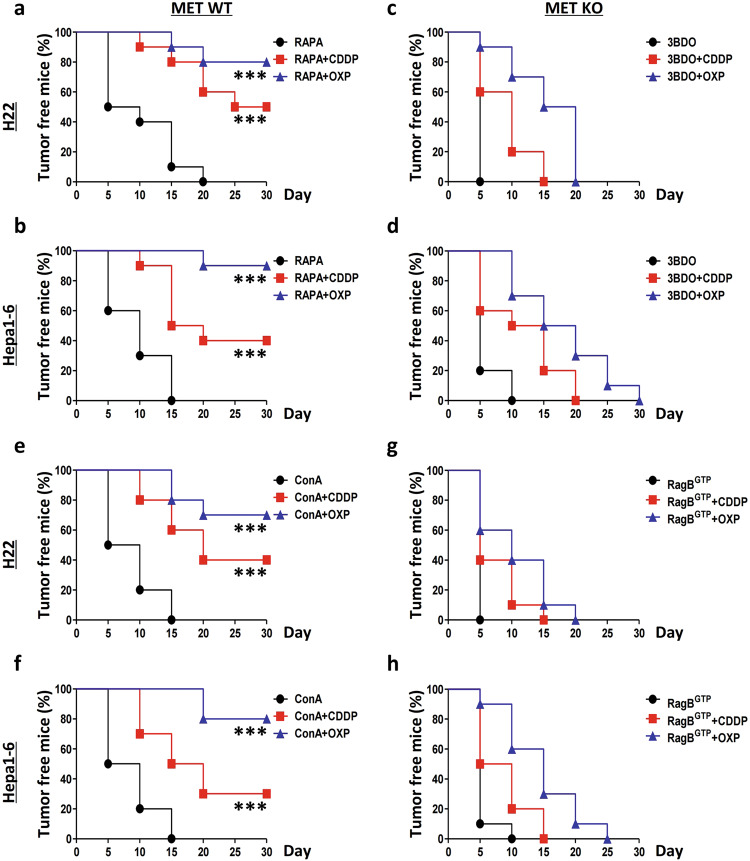
MET suppresses liver cancer vaccination through V-ATPase–MTOR signaling. **a**, **b** MTOR inhibition improves the protective efficacy of chemotherapy-based liver cancer vaccination. Wild-type (WT) H22 cells (**a**) and Hepa1-6 cells (**b**) were individually treated with rapamycin (RAPA) alone or in combination with cisplatin (CDDP) or oxaliplatin (OXP) and then subcutaneously (*s.c*.) injected into the left flank of C57BL/6 mice. One week later, all the mice were rechallenged with homologous untreated WT cells in the right flank. The tumor incidence is reported as Kaplan–Meier curves. Significance was determined by the means calculated with a likelihood ratio test. **c**, **d** MTOR activation suppresses MET deficiency-enhanced liver cancer immunogenicity. MET-deficient (MET-KO) H22 cells (**c**) and Hepa1-6 cells (**d**) were individually treated with 3BDO alone or in combination with CDDP or OXP, respectively, and subsequently experiments were conducted as previously described. **e**, **f** V-ATPase inhibition enhances the protective efficacy of chemotherapy-based liver cancer vaccination. WT H22 cells (**e**) and Hepa1-6 cells (**f**) were individually treated with concanamycin A (Con A) alone or in combination with CDDP or OXP, and then conducted as previously described. **g**, **h** Rag GTPase activation suppresses MET deficiency-enhanced liver cancer immunogenicity. MET-KO H22 cells (**g**) and Hepa1-6 cells (**h**) were individually transfected and treated with the RagB Q99L (RagB^GTP^) mutant alone or in combination with CDDP or OXP and subsequently conducted as before. *p-Value < 0.05, **p-value < 0.01, ***p-value < 0.001, ns (nonsignificant), compared to the indicated groups; *n* = 10 per group.

MTOR directly inhibits cancer immunity by promoting tumor expression of programmed death-ligand 1 (PD-L1) and thus creates a tumor microenvironment resistant to immune attack.^34,35^ Therefore, we investigated whether MET could suppress liver cancer immunogenicity through the MTOR signaling pathway. As expected, the MTOR inhibitor rapamycin (RAPA) enhanced chemotherapy-based vaccination induced by the WT H22 cells (80% for the OXP-treated cells and 50% for the CDDP-treated cells) (Fig. 7a) and Hepa1-6 cells (90% for the OXP-treated cells and 40% for the CDDP-treated cells) (Fig. 7b). However, the MTOR activator 3-benzyl-5-((2-nitrophenoxy) methyl)-dihydrofuran-2(3H)-one (3BDO) completely abrogated the protective efficacy of the chemotherapy-based vaccination induced by the MET-deficient H22 cells (0% for both the OXP- and CDDP-treated cells) (Fig. 7c) and Hepa1-6 cells (0% for both the OXP- and CDDP-treated cells) (Fig. 7d). Consistently, the V-ATPase inhibitor Con A also enhanced the chemotherapy-based vaccination induced by the WT H22 cells (70% for the OXP-treated cells and 40% for the CDDP-treated cells) (Fig. 7e) and Hepa1-6 cells (80% for the OXP-treated cells and 30% for the CDDP-treated cells) (Fig. 7f), similar to the results shown for RAPA. Furthermore, we observed that a RagB^GTP^ mutant completely repressed the protective efficacy of chemotherapy-based vaccination for the MET-deficient H22 cells (0% for both the OXP- and CDDP-treated cells) (Fig. 7g) and Hepa1-6 cells (0% for both the OXP- and CDDP-treated cells) (Fig. 7h), similar to 3BDO treatment. Hence, MTOR inhibitors can improve liver cancer immunogenicity.

Double-inhibition of MET and MTOR efficiently enhanced liver cancer vaccination in mice. Therefore, we investigated whether a combined treatment of MET and MTOR inhibitors can synergistically inhibit liver cancer cells *in vitro*. First, we analyzed the impacts of mTOR inhibition on MET-deficient cancer cells. WT and MET-KO HepG2 cells were individually treated with or without RAPA, and subsequently, the cell proliferation, viability, and colony formation capacity were analyzed. The results showed that, compared to that of the WT control, RAPA markedly inhibited the proliferation of the MET-KO cells (Supplementary Fig. S5a). Similar patterns were also observed for the assays of cell viability (Supplementary Fig. S5b) and colony formation (Supplementary Fig. S5c, d). Next, we used the TPRMET-NIH3T3 cell line to analyze the effects of the combined treatments of MET- and mTOR-targeted MET on MET-driven cancer cells. Intriguingly, we observed that, compared to the measures in the untreated vehicle control, the combination of MET inhibitor, capmatinib, and rapamycin significantly blocked cell proliferation (Supplementary Fig. S5e), viability (Supplementary Fig. S5f), and colony formation capacity (Supplementary Fig. S5g, h).

In conclusion, MET acted as a “gate-keeper” for suppressing liver cancer immunogenicity by regulating the lysosomal V-ATPase–mTOR complex. Specifically, HGF stimulates, whereas MET depletion or inhibition blocks, liver cancer immunogenicity (Supplementary Fig. S6a).

## Discussion

Various reports have suggested that MET affects tumor progression and survival under therapeutic stress through a complicated process, which partially explains the previous failures of drugs targeted to HGF–MET- signaling.^10–13,36–40^ Although MET is involved in neutrophil-mediated cancer immunity, contradictory conclusions regarding the pro- or anticancer effects of MET on neutrophils have largely limited its further exploitation and application in cancer immunotherapy.^41,42^ Moreover, Li et al. found that MET inhibitors stabilize PD-L1 to cause liver cancer cells to escape the body’s immune surveillance;^11^ in contrast, Martin et al. observed that MET inhibition reverses interferon γ-induced upregulation of PD-L1 expression levels in MET-amplified cancers.^13^ Although the effect of MET on cancer immunity is still unclear, in the present study, we revealed the importance of MET in the suppression of liver cancer immunogenicity, which is dependent on the MET–V-ATPase–MTOR pathway identified herein rather than on the traditional PI3K–AKT cascade. Thus, MET deficiency resulted in a marked enhanced efficacy of the chemotherapy-based anti-liver cancer vaccination. The outcomes of this study have widely expanded the targeting strategies of MET use in cancer prevention and therapy, especially for liver cancer.

Growth factor receptors (growth regulatory elements) are activated by corresponding ligands (sometimes multiple ligands), and they transduce outside signals for cellular proliferation, survival, and differentiation into cells.^43–45^ In contrast to specific nutrients, the deprivation of growth factors can neither promptly nor efficiently stimulate the activation of MTORC1 and its downstream signaling pathways. However, systematic signaling is coordinated among the growth factor–receptor(s) axis and the MTOR signaling pathway.^45,46^ For instance, both growth factors and MTOR signaling strongly inhibit autophagy and prevent insufficient energy conversion and unnecessary physical dissipation under normal conditions or metabolic stress.^47–49^ Nonetheless, the intracellular relationship between the growth factor–receptor axis and MTOR signaling pathway has not been fully understood (these interactions, if they exist, are likely established in the lysosome).^33,50^ For instance, at least in glioma, EGFR activates MTOR and its downstream substrate RPS6 through PKC, independent of AKT, which raises questions about the necessity of AKT as a critical intermediate for coupling the EGFR activation and MTOR signaling.^51^ In the current study, we also established that HGF enhances MET lysosomal translocation, where it interacts with ATP6V1A to regulate MTOR activation in an AKT-independent manner. Considering the dominant role of MTOR in metabolic processes, this finding may help us better understand MET-driven functions, especially in metabolism-controlled immunity, and may ultimately assist in formulating strategies for the development and use of MET-targeted therapies.

Another pivotal observation of this study suggests that the phosphorylation state of MET determines its level of interaction with the V-ATPase complex. To our knowledge, this is the first report showing an interaction between MET and the V-ATPase complex in a phosphorylation-dependent manner.^52,53^ However, the MET-binding motif was not specifically identified. MET substrates predominantly bind in proline-rich regions (PxPP).^27^ Unlike AKT (RXRXXS/T), AMPK (LXRXXS/T), and PKC [(R/K)(R/K)X(S/T)X(R/X)], the domains available to determine the potential substrates of MET are extremely limited.^54^ Therefore, more detailed studies are warranted to fully understand the association between MET and the V-ATPase complex.

## Supplementary information

Supplementary Material
