# Supplementary material for: The AKT-independent MET–V-ATPase–MTOR axis suppresses liver cancer vaccination
Source: Signal Transduct Target Ther. 2020 Aug 7;5:122. doi: 10.1038/s41392-020-0179-x (PMC7414041; doi:10.1038/s41392-020-0179-x)
Supplement: Supplementary file 1 — Supplementary Material [file 41392_2020_179_MOESM1_ESM.docx]

Supplementary Materials for

An AKT-independent MET–V-ATPase–MTOR axis suppresses liver cancer vaccination

Xing Huang, Xingyuan Xu, Xun Wang, Tianyu Tang, Enliang Li, Xiaozhen Zhang, Jian Xu, Hang Shen, Chengxiang Guo, Tao Xu, Jianhong Ren, Xueli Bai, Tingbo Liang

Correspondence to: [liangtingbo@zju.edu.cn (T.L.), shirleybai@zju.edu.cn (X.B.), or huangxing66@zju.edu.cn (X.H.)](mailto:xxxxx@xxxx.xxx)

**This PDF file includes:**

Materials and Methods

Figures. S1 to S6

Materials and Methods

Antibodies, chemicals, plasmids, and other reagents

Antibodies were obtained from the following sources: anti-MET (700261, Thermo Fisher, for WB; sc-161, Santa Cruz, for IP), anti-S6K1 (9202, Cell Signaling), anti-p-T389-S6K1 (9205, Cell Signaling), anti-ATP6V1A (17115-1-AP, Proteintech), anti-ATP6V1B (14780-1-AP, Proteintech), anti-ATP6V1D (14920-1-AP, Proteintech), anti-ATP6V1E (15280-1-AP, Proteintech), anti-ATP6V1F (17725-1-AP, Proteintech), anti-ATP6V1G (16143-1-AP, Proteintech), anti-ATP6V1H (26683-1-AP, Proteintech), anti-Flag (F1804, Sigma), anti-beta Actin (66009-1-Ig, Proteintech), anti-Rabbit IgG (HRP) (GTX221666-01, GeneTex), and anti-Mouse IgG (HRP) (GTX221667-01, GeneTex). Chemicals were obtained from the following sources: Cisplatin (S1166, Selleckchem), Oxaliplatin (S1224, Selleckchem), Afuresertib (S7521, Selleckchem), Capmatinib (S2788, Selleckchem), Concanamycin A (2656, Tocris Bioscience), Rapamycin (S1039, Selleckchem), and 3BDO (S8317, Selleckchem). Plasmids were obtained from the following sources: MET CRISPR/Cas9 KO Plasmid (h) (sc-400101, Santa Cruz), MET HDR Plasmid (h) (sc-400101-HDR, Santa Cruz), Met CRISPR/Cas9 KO Plasmid (m) (sc-421635, Santa Cruz), Met HDR Plasmid (m) (sc-421635-HDR, Santa Cruz). pRK5-HA GST RagB 99L (RagB^GTP^) was a gift from Prof. David Sabatini (Addgene plasmid #19303; http://n2t.net/addgene:19303; RRID: Addgene_19303), and pEGFP-Akt T308D/S473D (AKT-DD) was a gift from Prof. Julian Downward (Addgene plasmid #39536; http://n2t.net/addgene:39536; RRID: Addgene_39536). Other reagents were obtained from the following companies: Lipofectamine™ 3000 transfection reagent (L3000008, Invitrogen), UltraCruz® Transfection Reagent (sc-395739, Santa Cruz), phosphatase inhibitor cocktail (B15001, Bimake), protease inhibitor cocktail (B14001, Bimake), protein A/G-coupled agarose (sc-2003, Santa Cruz), anti-Flag gel (B23101, Bimake), ExpressPlus™ PAGE Gel (M42015C, GenScript), nitrocellulose membrane (66485, PALL), and SuperSignal™ West Femto Maximum Sensitivity Substrate (34095, Thermo Fisher).

Cell lines and cell culture

H22, Hepa1-6, HepG2, and HEK293T cell lines were obtained from the Cell Bank of the Chinese Academy of Sciences (Shanghai, China) or from KeyGEN BioTECH (Nanjing, China). KeyGEN BioTECH also performed mycoplasma contamination detection and short tandem repeat profiling for quality and identity verifications. All cell lines and derived cells were maintained at 37 °C in an incubator with 5% CO_2_ under standard conditions as specified by the respective suppliers. Except for amino acid starvation as indicated, cells were respectively cultured in Dulbecco’s modified Eagle’s medium or in Roswell Park Memorial Institute 1640 medium, supplemented with 2 mM L-glutamine, 1% NEAA, 100 units/ml penicillin, 100 mg/ml streptomycin and 10% FBS. No cell lines used in this work were misidentified, according to the database from the International Cell Line Authentication Committee (ICLAC). All cell lines were freshly thawed purchased seed cells, cultured for no more than 2 months, and regularly assessed by virtue of their morphological features to avoid cross-contamination or misuse.

Generation of CRISPR/Cas9-mediated knockout cell lines

As previously described with modification ^1,2^, for the generation of *MET* knockout cell lines, cells were individually co-transfected with either human MET CRISPR/Cas9 KO plasmid and MET HDR plasmid, or with mouse Met CRISPR/Cas9 KO plasmid and Met HDR plasmid. MET/Met CRISPR/Cas9 KO plasmids were designed so that they disrupt gene expression by causing a double-strand break in a 5' constitutive exon within the *MET/Met* gene. These MET/Met CRISPR/Cas9 KO plasmids consisted a pool of three plasmids, each encoding the Cas9 nuclease and a target-specific 20-nt guide RNA, which was designed for maximum knockout efficiency. The MET/Met HDR plasmid also consisted of a pool of two to three plasmids, each containing an HDR template, which corresponds to the cut sites that were generated by the MET CRISPR/Cas9 KO plasmid. Briefly, 1.5 × 10^5^ of cells were seeded in 3 ml of antibiotic-free standard growth medium per well using a 6-well tissue culture plate. When cell confluency had increased to 40%, a total of 2 µg of plasmids were transfected into cells according to the manufacturer's recommendation. Three days later, 1 µg/ml puromycin was added to a complete growth medium for selection of at least 10 days. After selection, cells were suspended, diluted, and re-seeded to ensure single clone formation. Knockout efficiency in every single clone was evaluated by Western blot with two different antibodies, and was further verified by sequencing the genomic DNA.

Immunoprecipitation and immunoblot

As previously described with modification ^1,3,4^, after rinsing three times with ice-cold PBS, cells were re-suspended in lysis buffer (25 mM HEPES, pH 7.5, 150 mM NaCl, 0.25% Triton X-100, 0.25% NP-40, 0.5% CHAPS, 10% glycerol, as well as a mixture of phosphatase inhibitors and protease inhibitors) on ice for 2 h, and then centrifuged at 14,000 × *g* for 20 min. The supernatants were pre-cleared with protein A/G-coupled agarose for 2 h, and subsequently subjected to incubation with 2.5 μg of the indicated antibodies or with 25 μl anti-Flag gel overnight at 4 °C. This incubation step was followed by addition of 25 μl protein A/G agarose for another 3 h to conjugate the antibody complex. After washing four times with lysis buffer, the immunoprecipitates were boiled in 1 × loading buffer for immunoblot analysis. Protein samples were further resolved by SDS-PAGE with a 4-20% 15-well ExpressPlus^TM^ PAGE Gel, and were then transferred onto a nitrocellulose membrane. Membranes were blocked with 10% BSA in TBST for 2 h, and subsequently incubated with indicated primary antibodies according to recommendations. After washing with TBST for three times, membranes were incubated with appropriate HRP-labeled secondary antibodies. Immunolabeling was developed with SuperSignal™ West Femto Maximum Sensitivity Substrate. Similar settings were applied for exposure time, brightness, contrast, and scanning conditions to capture parallel images.

Mass spectrometry

Informed by the results of the BCA assay, aliquots of 100 µg of each sample (in 1.5 ml centrifuge tubes) were used for peptide labeling with TMT 9-plex following the manufacturer's protocol (Pierce, TMT® Mass Tagging Kits and Reagent Kits). A high-performance liquid chromatography (HPLC) system (Diane NCS3500 system) was used with a mass range of 350 to 1600 m/z, and a Q Exactive mass spectrometer (Thermo Scientific) was applied for the identification and quantification of proteins by secondary mass spectrometry. Briefly, after reduction with 3 mM DTT for 45 min at 60 °C, samples were digested by trypsin overnight at 37 °C, desalted through ZipTip, and dried by rotary evaporator. The obtained immunoprecipitated samples were re-suspended in 96% H_2_O / 4% acetonitrile / 0.1% formic acid, and then subjected to LC-MS/MS. The top 20 multiply charged ions with the most intense signals were selected for secondary mass spectrum identification. Fragment ion spectra, produced via high-energy collision dissociation, were required for secondary mass spectrometry sequencing. The original data of mass spectrometry detection were quantitatively generated via the proportion of ions. The resolution of ions in MS-1 was 70,000 (at 400 m/z), and 17,500 in MS-2 (also at 400 m/z). Based on the proteome database (Human-RefSeq, NCBI), the obtained MS/MS spectra were further analyzed using the SEQUEST algorithm in Proteome Discoverer (version 1.3; Thermo Scientific) to identify separated peptides. Each survey scan was followed by automated sequential selection of seven peptides for CID, excluding previously selected ions.

Animal use and care

All animal research in this study was approved and supervised by the Institutional Animal Care and Use Committee (IACUC). All animal experiments strictly adhered to protocols, policies, and ethical guidelines formulated by the IACUC. NOD CRISPR *Prkdc Il2r-Gamma* triple-immunodeficient mice (NOD-*Prkdc^em26Cd52^Il2rg^em26Cd22^*/Nju (NCG), female, 5-week-old), had been created by sequential CRISPR-Cas9 editing of the *Prkdc* and *Il2rg* loci in the NOD/Nju mouse, and were obtained from the Nanjing Biomedical Research Institute of Nanjing University. C57BL/6 mice (female, 5-week-old) were obtained from the Comparative Medicine Center, Yangzhou University (Yangzhou, China). All mice were housed in specific pathogen-free conditions, and maintained at constant temperature and humidity under 12 h light-dark cycles, Mice received food and water *ad libitum*. After 3 weeks of the adaptive phase, these mice were used for the respective studies. None of the burden tumors exceeded the limit of neoplastic lesions (20% of mouse body mass or 20 mm in the longest axis) as a consideration of humane care.

Tumorigenesis assay

As previously described with modification ^1,5^, for tumorigenesis experiments, 1 × 10^5^ equal numbers of WT and MET KO H22, or 1 × 10^6^ equal numbers of Hepa1-6 cells were prepared in 100 μl PBS. These preparations were individually and subcutaneously (*s.c.*) inoculated into the right flank of 8-week-old female immunodeficient NCG mice or immunocompetent C57BL/6 mice. After inoculation, mice were monitored daily and weighed twice per week. When tumors became visible, tumor growth was routinely recorded at indicated times by caliper measurement, and tumor growth was monitored via tumor surface size (longest dimension × perpendicular dimension). Tumor weight was determined after scarification at the indicated day.

Immunogenicity assay

As previously described with modification ^5^, to assess tumor immunogenicity, paired WT and MET KO H22 or Hepa1-6 cells were individually transfected with constitutively activated mutants (RagB^GTP^, or AKT-DD) or vector controls. Forty-eight hours after transfection, cells were treated with Cisplatin (CDDP, 150 μM), Oxaliplatin (OXP, 150 μM), Concanamycin A (ConA, 5 μM), Rapamycin (RAPA, 0.5 μM), and 3BDO (50 μM), or vehicle control (equivalent volume) either alone or in combination for 24 h. The supernatants and detached cells were then collected and washed thrice to deplete the remnant drugs, and were subsequently inoculated *s.c.* into the left flanks of 6-week-old female C57BL/6 mice. Based on pre-tests, 1 × 10^5^ treated H22 cells, or 1 × 10^6^ treated Hepa1-6 cells were individually injected into 100 μl of PBS for each mouse. Seven days later, all mice were confirmed tumor-free in the vaccination flank, and un-transfected and/or untreated cancer cells with MET WT were further injected into the right flank of vaccinated and tumor-free mice. Tumor incidence in mice from the following day 5 was regularly monitored in n = 10 animals per group, and reported according to the Kaplan-Meier method until day 30. Animals were considered tumor-positive when a mass detected in the flank reached a size of at least 2 mm^2^ as calculated by the formula (size = longest dimension × perpendicular dimension) via caliper measurement. The non-occurrence of tumorigenesis indicated efficient antitumor vaccination.

Bioinformatics analysis

Multidimensional profiling datasets for 30 cancer types were collected from The Cancer Genome Atlas (TCGA, [http://cancergenome.nih.gov](http://cancergenome.nih.gov/)) database, and were subjected to bioinformatics analysis by two web portals: Tumor and Immune System Interaction Database (TISIDB, http://cis.hku.hk/TISIDB) ^6^ and Gene Expression Profiling Interactive Analysis (GEPIA2, <http://gepia2.cancer-pku.cn>) ^7^. The correlation between the relative abundance of 'Lymphocytes', 'MHCs', 'Immunomodulators', or 'Chemokines' and the expression of indicated genes were individually evaluated. FpClass ([http://dcv.uhnres.utoronto.ca/FPCLASS](http://dcv.uhnres.utoronto.ca/FPCLASS/)) ^8^ and String 10.5 ([https://string-db.org](https://string-db.org/)) databases were integrated to predict potential MET interactors and the protein-protein interaction network in LIHC.

Statistical analysis

No statistical methods were used to predetermine sample size, and no samples, mice, or data points were excluded from the reported analyses. Samples were not randomized to experimental groups, except for the allocations in specific mice experiments. The investigators were not blinded to allocation during experiments or outcome assessments. All images of Western blot are representatives of at least three independent experiments. All mice studies contained 10 individuals per group, and each of the total of 10 mice under the same treatment yielded similar results. Statistical analyses were performed using Microsoft Excel 2015 (Microsoft Corporation, Redmond, WA, USA) and GraphPad Prism 5 (GraphPad Software Inc., San Diego, CA, USA) to assess the differences between experimental groups. The variability within each group has been quantified from at least three technical or biological replicates, and data are presented as means ± SEM. Statistical significance was determined via the two-tailed Student’s *t*-test, one-way ANOVA analysis, or Likelihood ratio test. Spearman correlations, with a linear regression model, were conducted to evaluate the expression correlation in TCGA samples. * p-value < 0.05, ** p-value < 0.01, *** p-value < 0.001, as compared with negative, untreated, or scrambled control. p-value < 0.05 was considered to indicate statistically significant differences in this study.

References

1 Huang, X., Gan, G., Wang, X., Xu, T. & Xie, W. The HGF-MET axis coordinates liver cancer metabolism and autophagy for chemotherapeutic resistance. *Autophagy* **15**, 1258-1279, doi:10.1080/15548627.2019.1580105 (2019).

2 Su, Z. *et al.* Anti-MET VHH Pool Overcomes MET-Targeted Cancer Therapeutic Resistance. *Mol Cancer Ther* **18**, 100-111, doi:10.1158/1535-7163.MCT-18-0351 (2019).

3 Huang, X., Wu, Z., Mei, Y. & Wu, M. XIAP inhibits autophagy via XIAP-Mdm2-p53 signalling. *EMBO J* **32**, 2204-2216, doi:10.1038/emboj.2013.133 (2013).

4 Huang, X. *et al.* XIAP facilitates breast and colon carcinoma growth via promotion of p62 depletion through ubiquitination-dependent proteasomal degradation. *Oncogene* **38**, 1448-1460, doi:10.1038/s41388-018-0513-8 (2019).

5 Huang, X. *et al.* USP22 Deubiquitinates CD274 to Suppress Anticancer Immunity. *Cancer Immunol Res* **7**, 1580-1590, doi:10.1158/2326-6066.CIR-18-0910 (2019).

6 Ru, B. *et al.* TISIDB: an integrated repository portal for tumor-immune system interactions. *Bioinformatics* **35**, 4200-4202, doi:10.1093/bioinformatics/btz210 (2019).

7 Tang, Z., Kang, B., Li, C., Chen, T. & Zhang, Z. GEPIA2: an enhanced web server for large-scale expression profiling and interactive analysis. *Nucleic Acids Res* **47**, W556-W560, doi:10.1093/nar/gkz430 (2019).

8 Kotlyar, M. *et al.* In silico prediction of physical protein interactions and characterization of interactome orphans. *Nat Methods* **12**, 79-84, doi:10.1038/nmeth.3178 (2015).


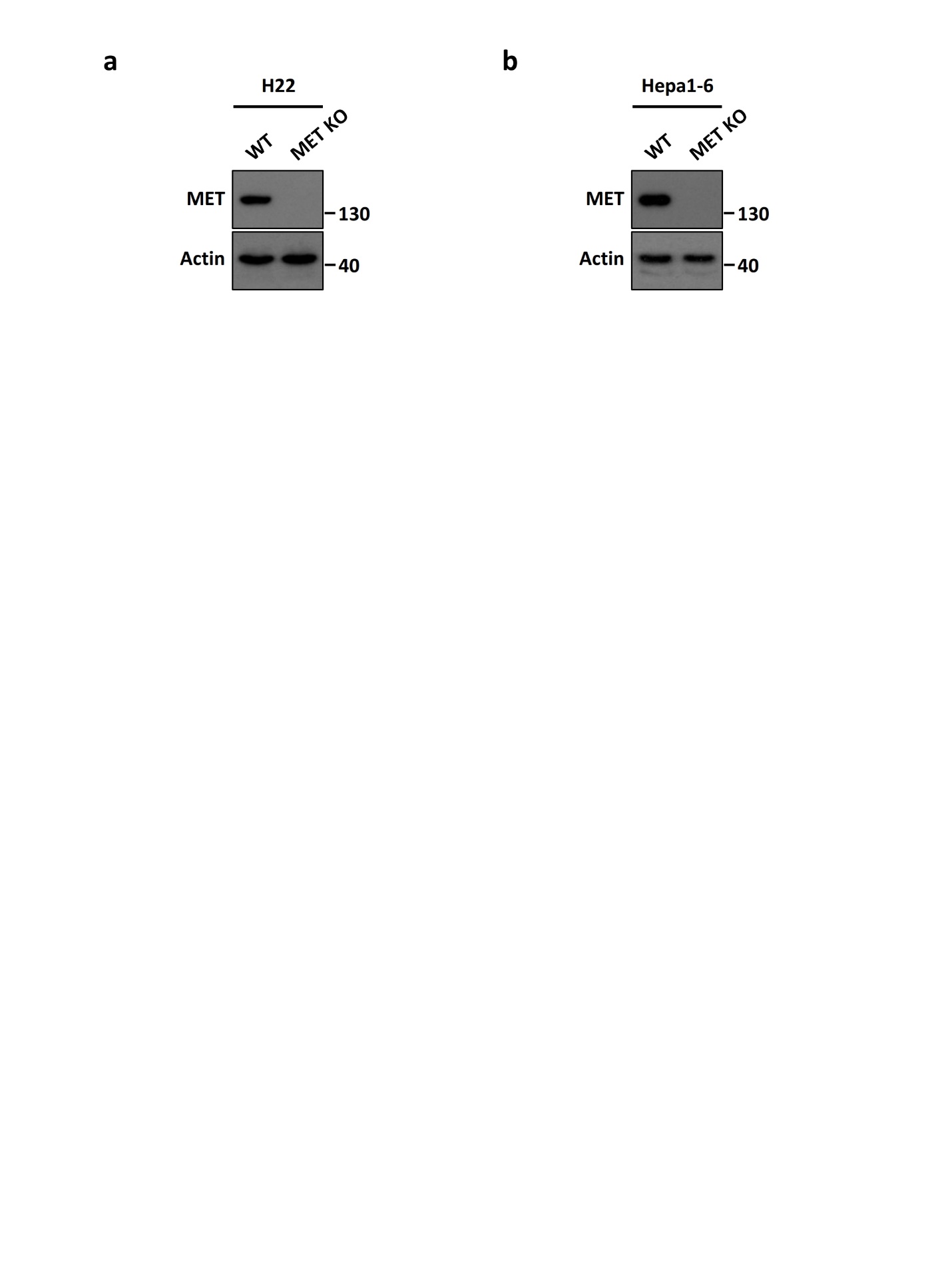


Figure. S1.

**Generation of MET-deficient** **murine liver cancer cell lines.** (**a**) Generation of MET-deficient H22 cell lines. WT and MET KO H22 cells were subjected to immunoblot analyses to assess knockout efficiency using antibodies as indicated. (**b**) Generation of MET-deficient Hepa1-6 cell lines. WT and MET KO Hepa1-6 cells were subjected to immunoblot analysis to assess knockout efficiency using antibodies as indicated.


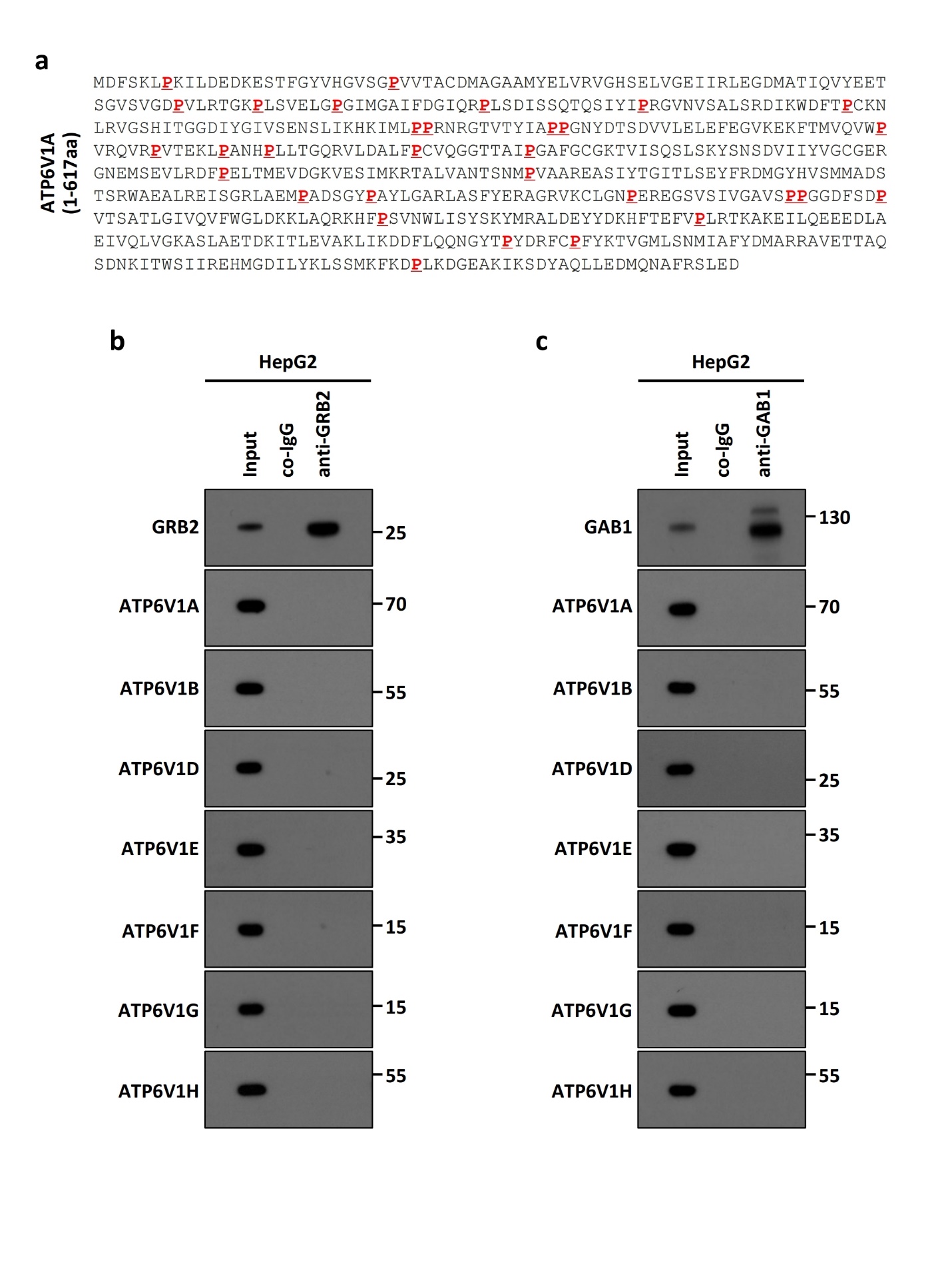


Figure. S2.

**MET interacts with the lysosomal V-ATPase complex in a non-canonical manner.** (**a**) No conserved MET binding motif was found in ATP6V1A. Detailed sequence analysis of ATP6V1A for potential MET binding site (PxPP) is shown as a representation. (**b-c**) No interaction between the V-ATPase complex and MET-associated scaffold protein GRB2 and GAB1. HepG2 cells (1 × 10^6^) were subjected to co-immunoprecipitation with anti-GRB2 antibody (**b**) or anti-GAB1 antibody (**c**). Immunoprecipitates and inputs were analyzed by immunoblot with antibodies as indicated.


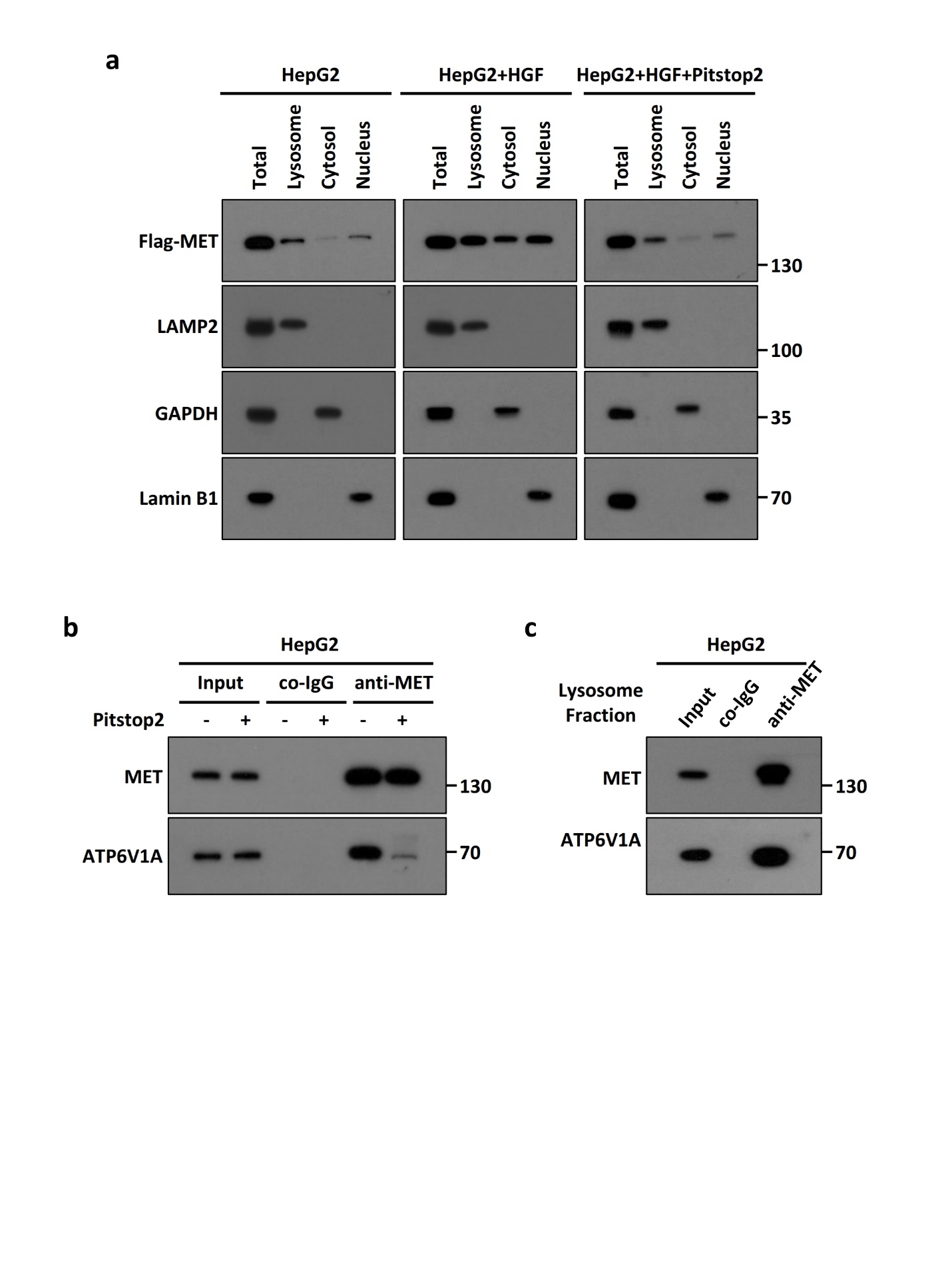


Figure. S3.

**MET binds to lysosomal V-ATPase through endocytosis.** (**a**) Inhibition of endocytosis blocks MET lysosomal translocation. HepG2 cells were serum-starved overnight and pretreated either with or without 30 μM Pitstop2 for 20 min. Cells were then stimulated either with or without 50 ng/ml HGF for 45 min, and subsequently subjected to fractionation assay with antibodies as indicated. (**b**) Inhibition of endocytosis blocks HGF-enhanced MET–ATP6V1A interaction. HepG2 cells (1 × 10^6^) were serum-starved overnight and pretreated either with or without 30 μM Pitstop2 for 20 min. Cells were then stimulated either with or without 50 ng/ml HGF for 45 min, and subsequently subjected to immunoprecipitation assay. (**c**) MET binds to ATP6V1A in lysosomes. The purified lysosomes (1 mg) from HepG2 cells were further subjected to both immunoprecipitation and immunoblot with antibodies as indicated.


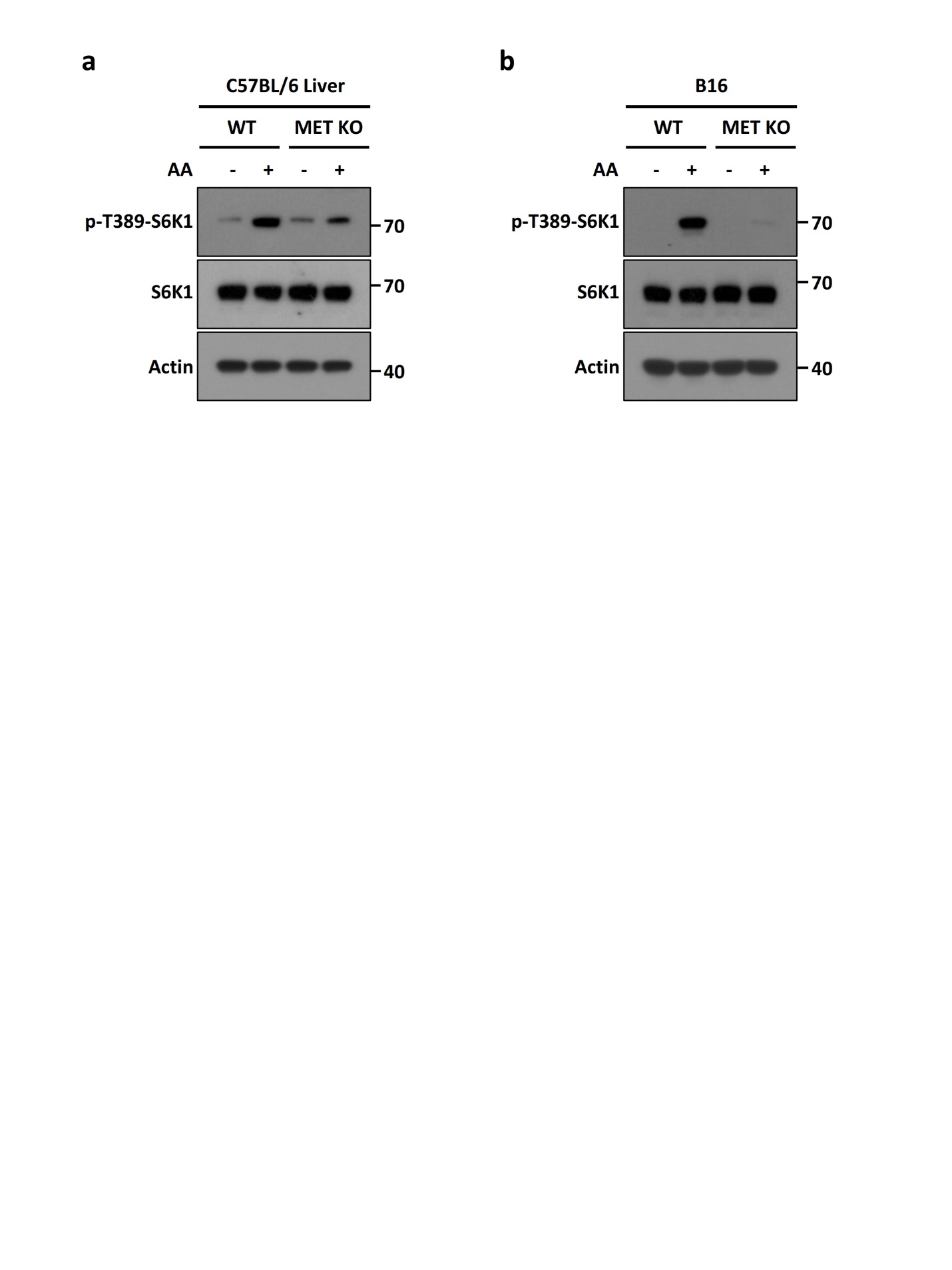


Figure. S4.

**The MET–V-ATPase–mTOR axis is a general regulatory mechanism.** (**a**) Amino acid-induced mTOR activation was restricted in Met deficient mice. WT and CRISPR/Cas9 system-mediated MET^Liver-KO^ C57BL/6 mice were starved for one day, and were then stimulated either with or without amino acids for 90 min by intravenous injection (*i.v*.). Then, mice were sacrificed, and liver lysates (40 μg) were subjected to immunoblotting with antibodies as indicated. (**b**) MET was required for amino acid-induced mTOR activation in epithelial cells. WT and MET KO B16 cells (5 × 10^4^) were individually deprived of amino acids for 90 min, and were then stimulated either with or without amino acids for 45 min, and subsequently subjected to immunoblot analysis.


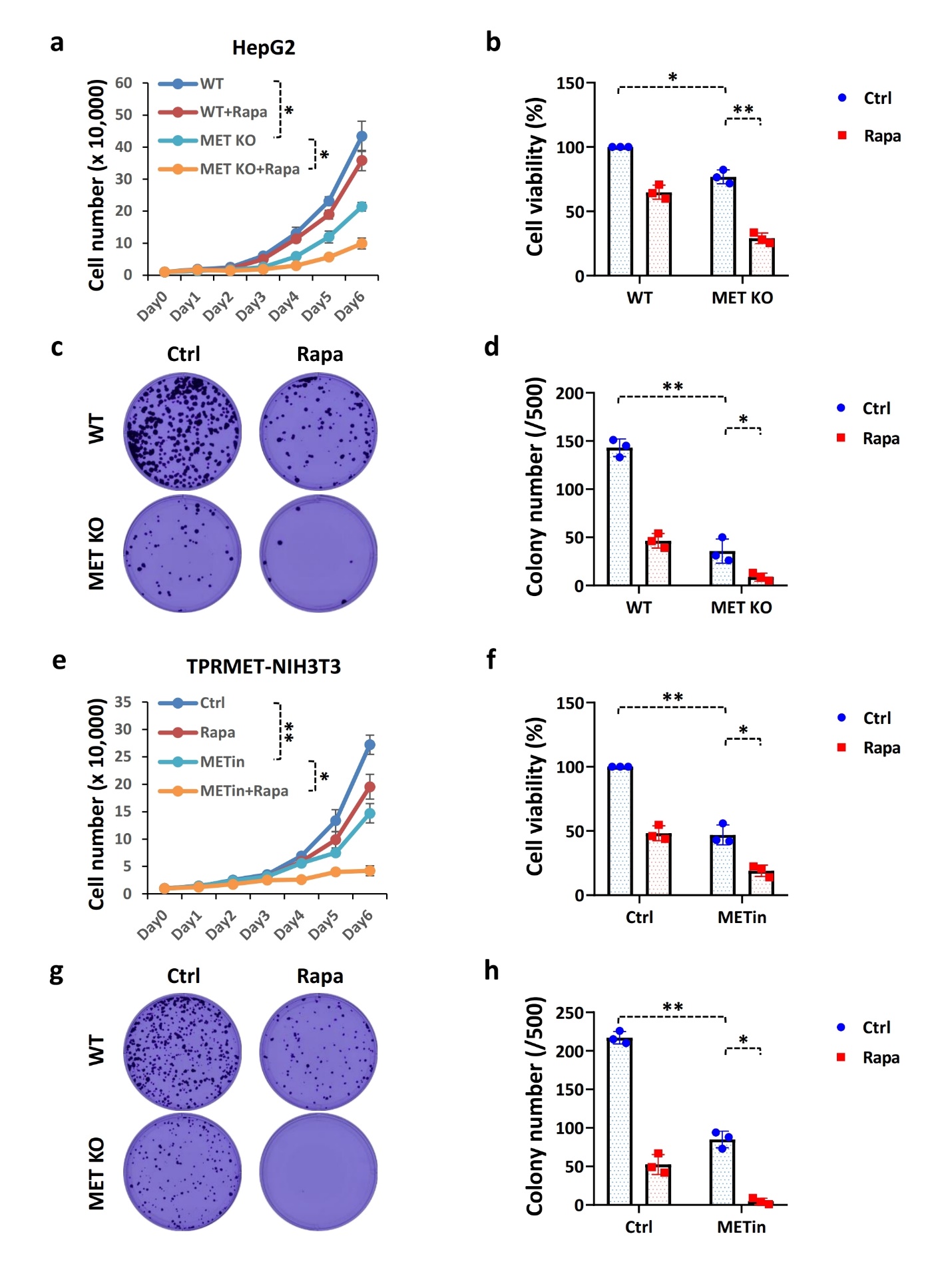


Figure. S5.

**The MET–V-ATPase–mTOR axis contributes to cancer cell proliferation, viability, and clonality *in vitro*.** (**a**) Impacts of the MET–V-ATPase–mTOR axis on cancer cell proliferation. WT and MET KO HepG2 cells (1 × 10^4^) were individually seeded overnight at day 0, and were subsequently treated with vehicle control (Ctrl) or 50 nM Rapamycin (Rapa), respectively. The growth medium was replaced every day. Cumulative cell numbers were counted at indicated times and are presented as growth curves. (**b**) Impacts of the MET–V-ATPase–mTOR axis on cancer cell viability. WT and MET KO HepG2 cells (2.5 × 10^3^) were individually seeded overnight, and were subsequently treated either with or without 50 nM Rapa, respectively, for 8 h. Then, cells were individually incubated with 0.25 mg ml^−1^ WST-8 solution at 37 °C for 2 h. The absorbance was measured at 450 nm to calculate the percentage of viable cells. (**c-d**) Impacts of the MET–V-ATPase–mTOR axis on cancer cell colony formation. WT and MET KO HepG2 cells (0.5 × 10^3^) were individually seeded overnight, and were subsequently treated either with or without 50 nM Rapa, respectively, for 2 weeks. The growth medium was replaced every 2 days. Then, cells were fixed with 4% cold PFA for 45 min, and then stained with 0.1% crystal violet for 2 h at room temperature. Representative images are shown as indicated (**c**), and colony numbers were quantified under a microscope (**d**). (**e**) Cancer cell proliferation targeting the MET–V-ATPase–mTOR axis. HepG2 cells (1 × 10^4^) were individually seeded overnight at day 0, and were subsequently treated with vehicle control (Ctrl), 10 nM Capmatinib (METin), or/and 50 nM Rapa, and were then subjected to cell proliferation analysis. (**f**) Cancer cell viability for targeting the MET–V-ATPase–mTOR axis. HepG2 cells (2.5 × 10^3^) were individually seeded overnight, and subsequently treated either with or without 10 nM METin, or/and 50 nM Rapa for 8 h, and were then subjected to cell viability analysis. (**g-h**) Cancer cell colony formation on targeting the MET–V-ATPase–mTOR axis. HepG2 cells (0.5 × 10^3^) were individually seeded overnight, and were subsequently treated either with or without 10 nM METin, or/and 50 nM Rapa for 2 weeks. Cells were then subjected to cell clonality analysis as described before. Data are presented as means ± s.d. from at least three independent experiments. Statistically significant differences (via two-tailed Student’s *t*-test) are marked as * (p < 0.05) or ** (p < 0.01).


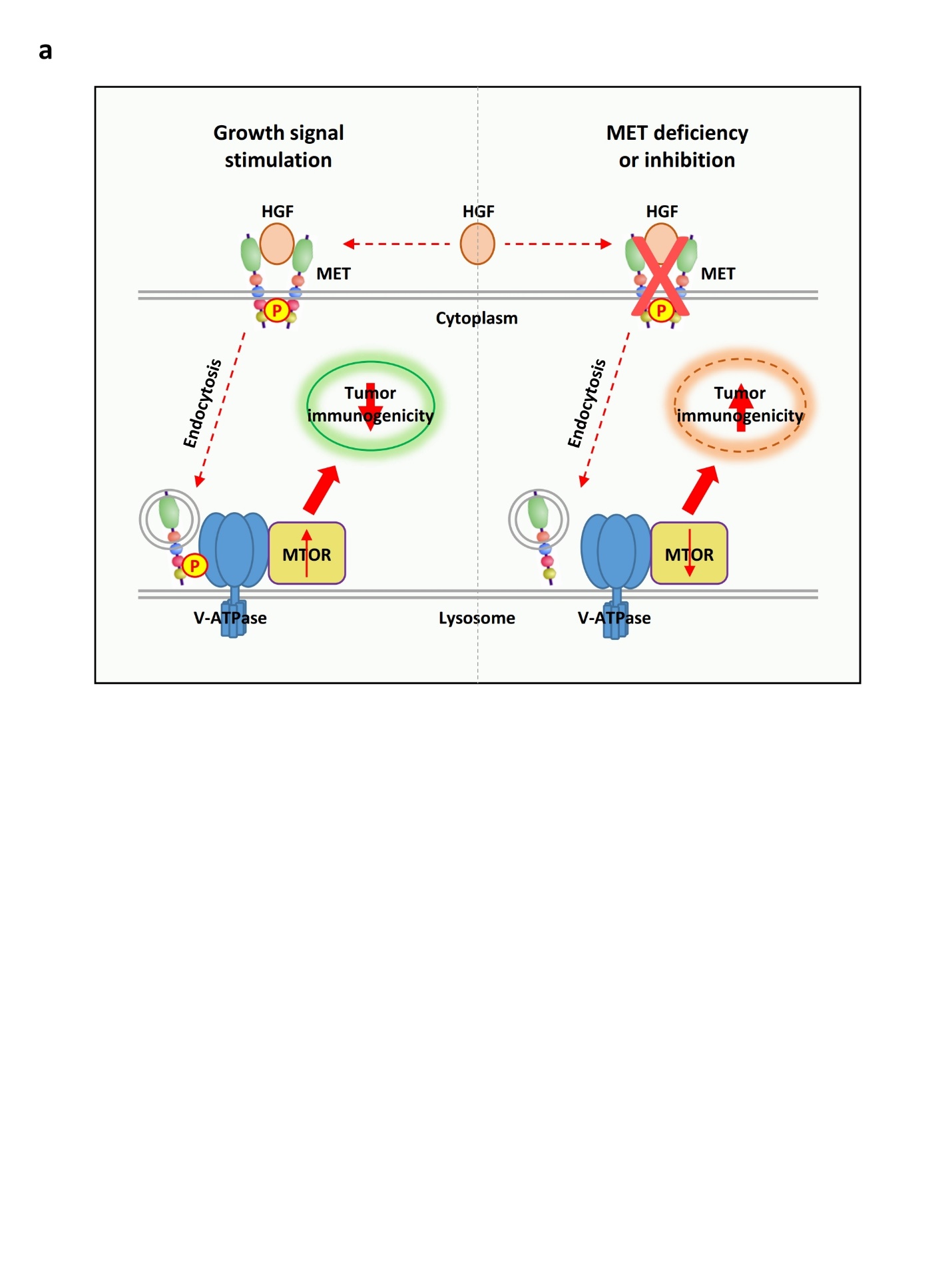


Figure. S6.

**Proposed model.** (**a**) A schematic model depicting that MET acts as a gate-keeper to suppress liver cancer immunogenicity by regulating the lysosomal V-ATPase–mTOR complex. HGF stimulates, while MET depletion or inhibition blocks this function.
